# Are *Helicobacter pylori* Infection and Fucoidan Consumption Associated with Fucoidan Absorption?

**DOI:** 10.3390/md18050235

**Published:** 2020-04-30

**Authors:** Makoto Tomori, Takeaki Nagamine, Masahiko Iha

**Affiliations:** 1 South Product Co. Ltd., Uruma 904-2234, Okinawa, Japan; miha.south@nifty.com; 2 Department of Nutrition, Takasaki University of Health and Welfare, Takasaki 370-0036, Gunma, Japan; nagamine-t@kendai-clinic.jp

**Keywords:** fucoidan, *Helicobacter pylori*, mozuku, *Cladosiphon okamuranus* Tokida, urinalysis

## Abstract

We examined the associations of *Helicobacter pylori* and mozuku consumption with fucoidan absorption. Overall, 259 Japanese volunteers consumed 3 g fucoidan, and their urine samples were collected to measure fucoidan values and *H. pylori* titers before and 3, 6, and 9 h after fucoidan ingestion. Compared to the basal levels (3.7 ± 3.4 ng/mL), the urinary fucoidan values significantly increased 3, 6, and 9 h (15.3 ± 18.8, 24.4 ± 35.1, and 24.2 ± 35.2 ng/mL, respectively) after fucoidan ingestion. The basal fucoidan levels were significantly lower in *H. pylori*-negative subjects who rarely ate mozuku than in those who regularly consumed it. Regarding the ΔMax fucoidan value (highest value − basal value) in *H. pylori*-positive subjects who ate mozuku at least once a month, those aged ≥40 years exhibited significantly lower values than <40 years old. Among subjects ≥40 years old who regularly consumed mozuku, the ΔMax fucoidan value was significantly lower in *H. pylori*-positive subjects than in *H. pylori*-negative ones. In *H. pylori*-positive subjects who ate mozuku at least once monthly, basal fucoidan values displayed positive correlations with *H. pylori* titers and ΔMax fucoidan values in subjects <40 years old. No correlations were found in *H. pylori*-positive subjects who ate mozuku once every 2–3 months or less. Thus, fucoidan absorption is associated with *H. pylori* infection and frequency of mozuku consumption.

## 1. Introduction

Fucoidan is a complex sulfated polysaccharide that is mostly found in brown marine algae. Fucoidan exhibits a broad spectrum of biological activities, including anti-inflammatory, immunomodulatory, anti-oxidant, anti-tumor, and anti-infection effects [1,2,3,4,5]. Several investigators have reported a potential role of fucoidan as an anti-*Helicobacter pylori* (*H. pylori*) agent based on its ability to disrupt the adhesion of the microbe to the gastric epithelium in vivo and in vitro [6,7,8,9]. The inhibitory effect of fucoidan derived from *Cladosiphon okamuranus* (Okinawa mozuku) on *H. pylori* was demonstrated in vitro by Shibata et al. [6]. Their study showed that the *H. pylori* binding to human gastric cell lines was inhibited more by *Cladosiphon* fucoidan than by fucoidan procured from *Fucus*. In addition, fucoidan blocked both Leb- and sulfatide-mediated attachment of *H. pylori* to gastric cells. They concluded that the inhibitory effect of *Cladosiphon* fucoidan on the binding of *H. pylori* and gastric cells might be caused by the coating with this component of the bacterial surface. However, no bacteriostatic or bactericidal activity was observed against *H. pylori* for any fucoidan preparation [9].

Fucoidan is reported to be absorbed across the intestinal tract via energy-dependent processes and pinocytosis [10,11,12]. In Japanese volunteers, fucoidan was detected in the majority of urine following oral administration [13]. Because the rate of fucoidan absorption through the small intestine was highly variable among the participants, various factors were suggested to influence its absorption. For example, the consumption of Okinawa mozuku (*Cladosiphon okamuranus* Tokida), a brown seaweed containing fucoidan, is an important factor associated with fucoidan absorption. Based on a previous report by Hehemann et al. [14], we speculated that the gastrointestinal microbiota can influence the absorption of fucoidan.

*H. pylori* is a Gram-negative, spiral-shaped, microaerophilic bacterium. It colonizes the entire gastric mucosa in approximately half of the world’s human population, and a poor socioeconomic condition is an important risk factor for infection [15,16,17,18]. *H. pylori* causes peptic ulcer disease and atrophic gastritis, and it is associated with primary gastric B-cell lymphoma and gastric adenocarcinoma. The host immune system cannot clear the infection, and it persists without treatment.

Many studies have focused on the modification of the gastric environment induced by *H. pylori* infection. For example, *H. pylori* infection can lead to the deficiency of vitamins, such as vitamin C, vitamin A, α-tocopherol, vitamin B_12_, and folic acid, as well as essential minerals [19,20,21]. Moreover, gastric *H. pylori* infection affects local and distant microbial populations and host responses.

Because fucoidan can bind to *H. pylori* and disrupt its attachment to the gastric epithelium [6,7,8], *H. pylori* infection is assumed to affect fucoidan absorption. In this study, we examined the effects of *H. pylori* infection on the absorption of fucoidan extracted from Okinawa mozuku in Japanese volunteers. Although fucoidan absorption is extremely low in humans, the fucoidan concentration after oral administration is approximately 10-fold higher in urine than in serum [22]. Therefore, urinary fucoidan concentrations were measured before and after the oral administration of mozuku fucoidan.

## 2. Results

### 2.1. Prevalence of H. Pylori Infection AccordingtTo the Frequency of Mozuku Consumption and Age

The relevance of mozuku consumption and age to *H. pylori* infection is shown in Table 1. Regarding age, *H. pylori* infection was detected in 60.0%, 58.7%, 61.9%, 77.8%, and 88.5% of participants aged 20–29, 30–39, 40–49, 50–59, and ≥60 years old, respectively.

According to logistic regression analysis, age was a significant risk factor for *H. pylori* infection, as the risk of infection was significantly higher in patients ≥40 years old than in those <40 years old. Mozuku consumption was not a significant risk factor for *H. pylori* infection (Table 2).

### 2.2. Urinary Fucoidan Values before and after Fucoidan Ingestion 

In all subjects, the urinary fucoidan values significantly increased 3, 6, and 9 h after fucoidan ingestion compared to the basal values (Table 3). The urinary fucoidan values were significantly higher at 6 and 9 h than those at 3 h. A significant difference was not observed in the urinary fucoidan values between the group that regularly ate mozuku and group that rarely ate mozuku.

### 2.3. Basal Levels (before Ingestion) of Fucoidan 

Among subjects who rarely ate mozuku, the basal fucoidan levels were significantly lower in the *H. pylori*-negative group than in the *H. pylori*-positive group. Among *H. pylori*-negative subjects, the basal fucoidan levels were significantly lower in those who rarely ate mozuku than in those who ate mozuku 1–3 times weekly, once monthly, or once every 2–3 months. Conversely, the basal fucoidan levels were not affected by the frequency of mozuku consumption or age in *H. pylori*-positive subjects (Table 4).

### 2.4. Relationship between H. pylori Titers and Basal Fucoidan Levels

**Figure 1:**
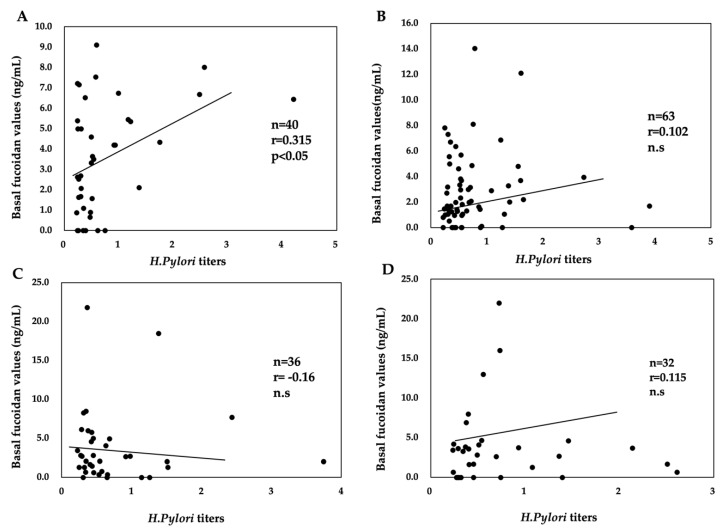
Relationship between *Helicobacter pylori* titers and basal fucoidan levels according to the frequency of mozuku consumption and age in among *H. pylori*-positive subjects. (**A**) Subjects aged <40 years who ate mozuku at least once a month. (**B**) Subjects aged ≥40 years who ate mozuku at least once a month. (**C**) Subjects aged <40 years who ate mozuku once every 2–3 months or less. (**D**) Subjects aged ≥40 years who ate mozuku once every 2–3 months or less. n = number of subjects, r = correlation coefficient, n.s = not significant.

Among *H. pylori*-positive subjects, a significant positive correlation existed between *H. pylori* titers and basal fucoidan levels in participants <40 years old who ate mozuku at least once monthly. A significant correlation between *H. pylori* titers and basal fucoidan levels was not found in participants aged ≥40 years irrespective of the frequency of mozuku consumption (Figure 1).

### 2.5. Maximum Absorption of Fucoidan (ΔMax Fucoidan Value)

Urinary fucoidan was detected in 252 of 259 subjects following a single oral dose of 3 g. The ΔMax fucoidan values exhibited a wide distribution, ranging from 0 to 273.6 ng/mL. Among the participants in whom urinary fucoidan was not detected, three rarely ate mozuku, one ate mozuku once every 2–3 months, and three ate mozuku once monthly.

Table 5 shows the relevance of *H. pylori* infection and age to the ΔMax fucoidan values. The ΔMax fucoidan values in all subjects were similar between *H. pylori*-positive and *H. pylori*-negative subjects. Compared with the values in *H. pylori-negative* subjects, the ΔMax fucoidan values of *H. pylori*-positive subjects tended to be higher in subjects in their 20s and 30s and lower in those in their 40s and 50s (data not shown). To determine relevance of age to fucoidan absorption, the subjects were divided into two age groups (<40 and ≥40 years).

ΔMax fucoidan values were significantly lower in subjects aged ≥40 years than in younger subjects among *H. pylori*-positive participants. No effect of age on ΔMax fucoidan values was observed among *H. pylori*-negative subjects. No significant difference of ΔMax fucoidan values was found according to the presence of *H. pylori* infection in either age group.

### 2.6. Relevance of H. Pylori Infection and Mozuku Consumption to Fucoidan Absorption

In a comparison between participants aged ≥40 years and those aged <40 years, the ΔMax fucoidan values were decreased by regular mozuku consumption in *H. pylori*-positive subjects but not in *H. pylori*-negative subjects (Table 6). Specifically, ΔMax fucoidan values were lower in *H. pylori*-positive subjects aged ≥40 years who ate mozuku at least once a month than in those who ate mozuku less frequently. Subsequently, the subjects were divided into groups based on the frequency of mozuku consumption, and the relevance of mozuku consumption to ΔMax fucoidan values was elucidated (Table 7).

Among *H. pylori*-positive subjects who ate mozuku at least once a month, ΔMax fucoidan values were significantly lower in those aged ≥40 years than in those aged <40 years. In addition, among *H. pylori*-positive subjects aged ≥40 years, the ΔMax fucoidan values were significantly lower in those who regularly consumed mozuku than in those who rarely ate mozuku. In addition, the ΔMax fucoidan values were significantly different between *H. pylori*-positive (16.8 ± 20.8) and *H. pylori*-negative subjects (32.1 ± 17.6) among those who ate mozuku at least once a month. However, no difference in ΔMax fucoidan values was noted according to age or frequency of mozuku consumption among *H. pylori*-negative participants.

### 2.7. Relationship between the Basal and Δmax Fucoidan Values in H. Pylori-Positive Subjects

**Figure 2:**
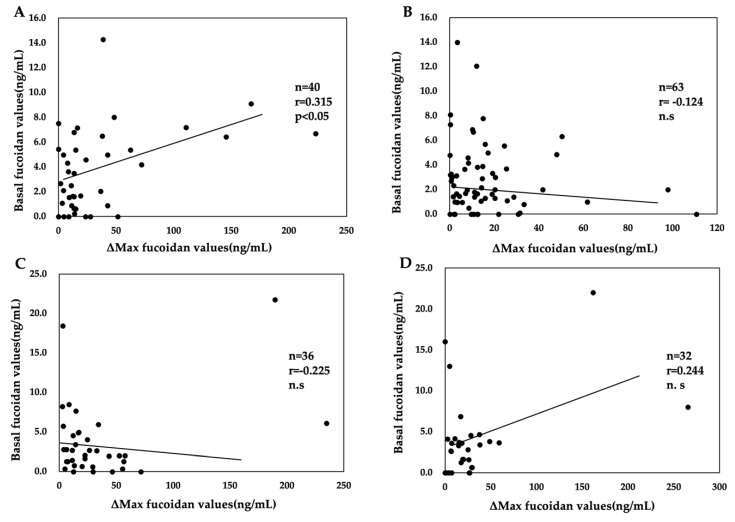
Relationship between basal fucoidan values and the maximum absorption of fucoidan (ΔMax fucoidan values) according to the frequency of mozuku consumption and age in *Helicobacter pylori*-positive subjects. (**A**) Consumption of mozuku at least once monthly among subjects aged <40 years (**B**) Consumption of mozuku at least once monthly among subjects aged ≥40 years (**C**) Consumption of mozuku once every 2–3 months or less among subjects aged <40 years (**D**) Consumption of mozuku once every 2–3 months or less among subjects aged ≥40 years. n = number of subjects, r = correlation coefficient, n.s = not significant.

Among *H. pylori*-positive subjects who regularly consumed mozuku, a significant positive correlation between the basal and ΔMax fucoidan values was found for those aged <40 years but not those aged ≥40 years. No significant correlation was found between the basal and ΔMax fucoidan levels among participants who rarely ate mozuku (Figure 2).

In addition, no significant correlations were found between the basal and ΔMax fucoidan levels in *H. pylori*-negative subjects regardless of the frequency of mozuku consumption (data not shown).

## 3. Discussion

The present study revealed an association between *H. pylori* infection and the absorption of fucoidan. Specifically, fucoidan absorption was significantly diminished among *H. pylori*-positive subjects aged ≥40 years who ate mozuku at least once monthly, whereas no association was found among *H. pylori*-negative subjects irrespective of the frequency of mozuku consumption and age. In addition, fucoidan absorption was not diminished among *H. pylori*-positive subjects who ate mozuku once every 2–3 months or less; therefore, mozuku consumption affects the absorption of fucoidan. Although the precise mechanisms by which *H. pylori* infection and mozuku consumption reduce fucoidan absorption have not been determined, a few possibilities have been postulated.

As ΔMax fucoidan values were similar between *H. pylori*-negative and *H. pylori*-positive subjects among participants aged ≥40 years, *H. pylori* was less likely to directly diminish the absorption of fucoidan in this age group. Excluding *H. pylori*-positive participants aged ≥40 years who ate mozuku regularly, ΔMax fucoidan values were similar between *H. pylori*-positive and *H. pylori*-negative subjects. In addition, the frequency of mozuku consumption among *H. pylori*-positive participants was similar between subjects aged <40 years and those aged ≥40 years; therefore, the frequency of mozuku consumption is not directly associated with the absorption of fucoidan. Given that *H. pylori* positivity and regular mozuku consumption were associated with diminished fucoidan absorption among subjects aged ≥40 years but not among younger subjects, the duration of *H. pylori* infection and the frequency of mozuku appear important for fucoidan absorption.

How do frequent mozuku consumption and *H. pylori* infection disturb fucoidan absorption in subjects aged ≥40 years? *H. pylori* can change the secretion and acidification functions of the stomach because it penetrates into this organ. Although nutrient absorption does not occur in the stomach, *H. pylori* infection can affect the digestion and absorption of nutrients such as vitamin B_12,_ vitamin C, vitamin A, vitamin E, and folate [19,20,21]. Shibata et al. [7] reported that mozuku fucoidan can bind to *H. pylori* and inhibit its attachment to the gastric mucosa at pH 2.0 and 4.0, but not at pH 7.4. It is well known that *H. pylori* rarely causes atrophic gastritis in young people (<40 years old), whereas *H. pylori*-induced atrophic gastritis tends to be rather common in the elderly [15,16,17,18]. When hypochlorhydria occurs after *H. pylori*-induced atrophic gastritis, intragastric pH increases, consequently inhibiting the ability of *H. pylori* to bind to fucoidan. However, fucoidan absorption was not diminished in *H. pylori*-positive subjects aged ≥40 years who rarely ate mozuku, suggesting the influence of a long duration of mozuku ingestion on fucoidan absorption. Amornlerdpison et al. reported that fucoidan present in mozuku acts as an antagonist of the H_2_ receptor (similar to cimetidine), decreasing the acidity of gastric acid and raising the pH in the stomach [23]. Taken together, *H. pylori*-induced atrophic gastritis and a long duration of mozuku ingestion may markedly decrease acid secretion, consequently leading to the failure of *H. pylori* to bind fucoidan and reductions of its absorption in the small intestine in people aged ≥40 years. Thus, the possible mechanism by which *H. pylori* infection leads to reduced gastric acid secretion and fucoidan absorption has not been sufficiently investigated to draw definite conclusions, and other mechanisms other than hypochlorhydria following *H. pylori* infection are possible.

Of note, significant positive correlations of the basal fucoidan levels with both *H. pylori* titers and ΔMax fucoidan values were revealed in *H. pylori*-positive subjects aged <40 years who frequently consumed fucoidan. Such correlations were not found in the corresponding group of participants aged ≥40 years, nor were they observed in *H. pylori*-positive subjects who rarely ate mozuku or in *H. pylori*-negative subjects. Because the significance of the positive correlation observed in *H. pylori*-positive subjects aged <40 years who frequently consumed fucoidan is unclear, further research is necessary to clarify the relevance of *H. pylori* infection and mozuku intake to fucoidan absorption using a large number of subjects.

Interestingly, basal fucoidan levels were significantly increased by *H. pylori* infection and mozuku consumption. As *H. pylori* is known to affect the absorption of various nutrients, this stomach bacterium may participate in basal fucoidan absorption. In recent years, “the nutrition-gut microbiome-physiology axis” has attracted substantial attention [24,25,26,27,28]. Because *H. pylori* can induce drastic alterations in the variety of the gastrointestinal microbiota [29,30,31], the microbe is speculated to increase basal fucoidan levels by modifying the gastrointestinal microbiota. Basal fucoidan levels were also significantly higher in *H. pylori*-negative subjects who regularly consumed mozuku than in their counterparts who rarely ate mozuku, which confirmed our previous findings [13]. We speculated that Japanese people may have acquired digestive enzymes from mozuku because the seaweed is extensively consumed within this area. Because of the limited evidence, the overall significance of *H. pylori* infection and mozuku consumption to basal fucoidan levels is unclear.

This study had several limitations. First, subjects who received eradication therapy for *H. pylori* and underwent gastrectomy prior to the study were not excluded. The study also did not exclude subjects who used complementary and alternative medicines, which can affect the absorption of fucoidan.

Second, a urine-based ELISA kit (URINELISA) was used to assay *H. pylori* infection, and the high accuracy of this test was certified by several investigators [32,33]. A disadvantage of this test is that proteinuria can cause false-positive results; therefore, urine protein levels should be measured in future research. We plan to study the relationship of *H. pylori* infection with fucoidan absorption using serum-based ELISA kits or the ^13^C urea breath test (^13^C-UBT) in future research.

In addition, the specificity of our fucoidan ELISA was limited. We assayed urinary fucoidan levels using a polyclonal antibody for Okinawa mozuku fucoidan, which weakly cross-reacted with *Fucus vesiculosus* fucoidan [22]. Because the brown seaweeds of kombu (*Laminaria japonica*) and wakame (*Undaria pinnatifida*) are traditional foodstuffs in Japan, fucoidan contained in these seaweeds may cross-react with our antibody. Further studies are necessary to elucidate the effects of mozuku consumption on the intestinal absorption of fucoidan using ELISA with a monoclonal antibody.

## 4. Materials and Methods

### 4.1. Subjects

We published pamphlets describing the purpose, methods, and exclusion items of our research titled “The reference of *H. pylori* infection to absorption of mozuku fucoidan” on the Internet and recruited volunteer participants. Two hundred sixty-two Japanese people submitted applications from April 2014 to June 2016. They completed a questionnaire assessing gender, age, and mozuku consumption. We enrolled 259 volunteers who completed questionnaires and collected urine samples as planned. Subjects were divided into five age groups: 20–29, 30–39, 40–49, 50–50, and ≥60 years old.

The frequency of mozuku consumption was divided into five groups as follows: approximately 1–3 times weekly, approximately once every 2 weeks, approximately once monthly, approximately once every 2–3 months, and rarely (Table 1).

This study was conducted according to the Declaration of Helsinki. The protocol of the study was approved by the Ethics Committee of South Product Co., Ltd. (UMIN000039117). Following an explanation of the study and its aim, all subjects provided informed consent.

### 4.2. Oral Intake of Fucoidan and Collection of Urine Samples

Subjects refrained from marine algae and fucoidan supplementation on the day before the test and on the day of the test to avoid the effects of diet. Subjects orally consumed two fucoidan drinks (1500 mg/bottle) at 9:00 in the morning. Urine samples were extracted four times, namely, before (0) and 3, 6, and 9 h after fucoidan ingestion. Urine samples were collected by a parcel delivery service.

In this study, the subjects orally consumed 3 g of mozuku fucoidan. The drink was prepared by South Product Co., Ltd.

### 4.3. Assay for Fucoidan Levels in Urine Samples

Urine fucoidan levels were assayed using a sandwich ELISA method developed by our laboratory [22]. The reproducibility of the fucoidan ELISA method was as follows. The intra- and inter-assay CVs for serum, plasma, and urine, using high and low concentrations of fucoidan, were in the range of 1.5–13.4%. The detection limit concentration of our ELISA was less than 1 ng/mL.

### 4.4. Assay for Anti-H. pylori Antibody Titers in Urine

Single-void urine samples were obtained and stored at 2–8 °C until use. Urinary IgG antibodies to *H. pylori* were measured using a urine-based ELISA kit (URINELISA^®^, Otsuka Pharmaceutical Co., Ltd.) that utilizes a VacA- and CagA-positive *H. pylori* strain isolated from a Japanese patient with gastritis as the antigen source. This ELISA-based test result was considered positive when a cutoff index of 1.0 (optical density = 0.218) or greater was obtained after measurement of the optical density according to the manufacturer’s instructions [32,33,34].

The ΔMax fucoidan value was calculated by subtracting the basal value (before ingestion) from the highest level of urinary fucoidan following fucoidan ingestion. If the basal fucoidan level was higher than that after fucoidan ingestion, then the ΔMax fucoidan value was recorded as 0.

### 4.5. Statistical Analysis

The urinary fucoidan values after fucoidan ingestion were analyzed using two-way analysis of variance (ANOVA) or one-way ANOVA followed by Tukey’s test for multiple comparisons. SAS version 9.4 (Statistical Analysis Software 9.4, SAS Institute Inc., Cary, NC, USA) was used to perform statistical analyses.

The Mann–Whitney U-test was used to analyze between-group differences. Statistical correlations were analyzed using Spearman’s rank correlation coefficient. In addition, multiple regression analysis was performed with *H. pylori* infection as the dependent variable and age and mozuku consumption as the independent variables. The results were expressed as the hazard ratio and 95% confidence interval. Data are expressed as the mean ± SD. *P* < 0.05 indicated a statistically significant difference.

## 5. Conclusions

The present data illustrated that fucoidan absorption is associated with *H. pylori* infection and mozuku consumption. Fucoidan absorption in *H. pylori*-positive subjects who regularly consumed mozuku differed by age, being significantly lower in subjects aged ≥40 years than in their younger counterparts. A significant positive correlation between the basal fucoidan level and ΔMax fucoidan value was found among subjects aged <40 who regularly consumed mozuku but not among their older counterparts. Further studies are needed to elucidate the precise mechanisms influencing fucoidan absorption.

## Figures and Tables

**Table 1:** *Helicobacter pylori* infection according to the frequency of mozuku consumption and age.

Age Group	1–3 Times Weekly		Once Every 2 Weeks		Once Monthly		Once Every 2–3 Months		Hardly Eat	
	*H. Pylori*		*H. Pylori*		*H. Pylori*		*H. Pylori*		*H. Pylori*	
	(-)	(+)	(-)	(+)	(-)	(+)	(-)	(+)	(-)	(+)
20’s (n = 50)	2	5	6	7	5	5	4	5	3	8
30’s (n = 75)	2	3	3	10	6	11	10	11	9	10
40’s (n = 63)	3	4	2	10	6	11	10	7	3	7
50’s (n = 45)	2	6	2	7	2	11	3	4	1	7
≥60’s (n = 26)	0	6	0	2	2	7	1	5	0	3

20’s: 20–29 years old, 30’s: 30–39 years old, 40’s: 40–49 years old, 50’s: 50–59 years old, ≥60’s: over 60 years old. n = number of subjects.

**Table 2:** Relevance of age and mozuku consumption to *Helicobacter pylori* infection: logistic regression analysis.

		Odds Ratio	95%CI
**Habit of eating mozuku**		1.12	0.89–1.42
Age	40y.o.<	1.00	1.01–2.85
	≥40y.o.	1.70	

y.o.: years old.

**Table 3:** Time course of urinary fucoidan.

	0	3 h	6 h	9 h
	ng/mL			
Subjects (n = 259)	3.7 ± 3.4	15.3 ± 18.8 ^a^	24.4 ± 35.1 ^a,b^	24.2 ± 35.2 ^a,b^

All data are presented as mean ± SD. Different letters indicate a significant difference as follows: ^a^ compared to the basal value (*p* < 0.01). ^b^ Compared to the fucoidan values at 3 h (*p* < 0.01). n = number of subjects.

**Table 4:** Basal fucoidan levels according to the frequency of mozuku consumption in *Helicobacter pylori*-negative and *Helicobacter pylori*-positive subjects.

Habit of Eating Mozuku	*H. pylori* (-)	*H. pylori* (+)	*P-Value* *H. pylori (-) vs H. pylori (+)*
1–3 times weekly	4.1 ± 1.3 (n =9) ^a^	3.2 ± 3.0 (n = 24)	0.29
Once every 2 weeks	2.7 ± 3.2 (n = 14)	3.2 ± 2.6 (n = 35)	0.58
Once monthly	3.1 ± 3.0 (n = 21) ^b^	2.8 ± 2.8 (n = 44)	0.69
Once every 2–3 months	3.3 ± 3.3 (n = 28) ^c^	4.3 ± 5.7 (n = 33)	0.40
hardly eat	1.4 ± 1.5 (n = 16)	3.3 ± 3.4 (n = 35)	0.01

All data are presented as mean ± SD. Different letters indicate a significant difference as follows: ^a^ compared to *H. pylori*-negative subjects who hardly ate mozuku (*p* = 0.01); ^b^ compared to *H. pylori*-negative subjects who hardly ate mozuku (*p* = 0.03); ^c^ compared to *H. pylori*-negative subjects who hardly ate mozuku (*p* = 0.03). n = number of subjects.

**Table 5:** Comparison of ΔMax fucoidan values by age.

	*H.pylori* (-)	*H.pylori* (+)	*H.pylori* (-) vs *H.pylori* (+)
Total	29.4 ± 40.1 (n = 88)	24.2 ± 37.1 (n = 171)	*P* = 0.300
40 y.o.<	26.4 ± 38.8 (n = 52)	35.3 ± 47.6 (n = 76)	*P* = 0.323
≥40 y.o.	33.8 ± 59. 8 (n = 36)	21.9 ± 35.3 (n = 95) ^a^	*P* = 0.135

All data were presented as mean ± SD. ^a^ There is a significant difference (*p* < 0.01) compared to H. pylori-positive subjects aged <40 years. n = number of subjects, y.o.: years old; *P* = *P* value.

**Table 6:** Relevance of *Helicobacter pylori* infection, frequency of mozuku consumption, and age to ΔMax fucoidan values.

Habit of Eating Mozuku	*H.pylori* (-)		*H.pylori* (+)		*P*-Value *H. pylori*(+) Aged <40 y.o. vs. *H. pylori*(+) Aged ≥40 y.o.
	40 y.o.<	≥40 y.o.	40 y.o.<	≥40 y.o.	
**1–3 times weekly**	25.0 ± 8.2 (n = 4)	37.8 ± 20.3 (n = 5)	20.4 ± 16.1 (n = 8)	17.4 ± 25.6 (n = 16)	n.s
Once every 2 weeks	24.5 ± 25.2 (n = 10)	36.4 ± 14.8 (n = 4) ^a^	34.8 ± 52.7 (n = 17)	12.9 ± 14.7 (n = 18)	0.08
Once monthly	29.1 ± 71.6 (n = 11)	27.7 ± 17.8 (n = 10)	41.9 ± 54.5 (n = 16)	18.9 ± 21.4 (n = 28)	0.06
Once every 2-3 months	31.1 ± 31.8 (n = 15)	39.3 ± 67.0 (n = 13)	36.2 ± 46.7 (n = 18)	42.5 ± 73.1 (n = 15)	n.s
hardly eat	24.4 ± 22.0 (n = 12)	15.8 ± 22.9 (n = 4)	38.1 ± 53.3 (n = 18)	22.2 ± 17.9 (n = 17)	n.s

All data are presented as mean ± SD. ^a^ Compared to H. pylori-negative subjects aged ≥40 years who hardly ate mozuku (*p* < 0.01). n = number of subjects, n.s: not significant; y.o.: years old.

**Table 7:** Relevance of *Helicobacter pylori* infection and age to ΔMax fucoidan values according to the frequency of mozuku consumption.

Habit of Eating Mozuku	*H.pylori* (-)		*H.ylori* (+)	
	40 y.o <	≥40 y.o	40 y.o <	≥40 y.o
Regularly consumed mozuku ^1)^	26.6 ± 48.7 (n = 25)	32.1 ± 17.6 (n = 19)	34.5 ± 48.1 (n = 40)	16.8 ± 20.8 ^a,b,c^ (n = 63)
Rarely ate mozuku ^2)^	28.1 ± 27.6 (n = 27)	33.8 ± 59.8 (n = 17)	33.9 ± 47.7 (n = 36)	30.5 ± 51.9 (n = 32)

All data are presented as mean ± SD. ^1)^ Regularly consumed mozuku: 1–3 times weekly + once every 2 weeks + once monthly; ^2)^ rarely ate mozuku: once every 2–3 months + hardly ate. ^a^ Compared to *H. pylori*-positive subjects aged <40 years who ate mozuku at least once monthly (*p* = 0.03). ^b^ Compared to *H. pylori*-positive subjects aged ≥40 years who ate mozuku once every 2–3 months or less (*p* = 0.01). ^c^ Compared to *H. pylori*-negative subjects aged ≥40 years who ate mozuku at least once monthly (*p* = 0.01). n = number of subjects, y.o = years old.
